# Familial Adenomatous Polyposis: Case Report and Literature Review

**DOI:** 10.7759/cureus.31609

**Published:** 2022-11-17

**Authors:** Juan José Chaves, Viviana Chaves-Cabezas, Rafael Parra-Medina, José Octavio Chaves-Chamorro

**Affiliations:** 1 Department of Pathology, Fundación Universitaria de Ciencias de la Salud, Hospital San José, Bogota, COL; 2 Department of Pathology, Fundación Universitaria de Ciencias de la Salud, Hospital San José, Bogotá, COL; 3 Department of Pathology, Instituto Nacional de Cancerología, Bogotá, COL; 4 Department of Gastroenterology, Hospital Civil de Ipiales, Ipiales, COL

**Keywords:** genetic syndromes, colombia, colorectal cancer, family research, familial adenomatous polyposis

## Abstract

Familial adenomatous polyposis (FAP) is an autosomal inheritance disease characterized by 100 or more adenomatous polyps in the colon and rectum with a high risk of developing colorectal carcinoma (CRC). The management of this disease is based on early screening and timely follow up, with subsequent planning of risk-reducing or therapeutic surgeries. We present a case of a patient with a strong family history of FAP with a “de novo” diagnosis of CRC. Furthermore, a literature discussion of current and future perspectives of treatment is performed.

## Introduction

Familial adenomatous polyposis (FAP) is a rare genetic disease with autosomal inheritance caused predominantly by germline mutations in the adenomatous polyposis coli (APC) gene, with a family history in 70-80% of cases [1]. Clinical diagnosis of the “classical FAP” is made with 100 or more adenomatous polyps in the colon and rectum or fewer than 100 polyps with at least one family history of confirmed FAP [2]. The development of colorectal carcinoma (CRC) in untreated patients is almost 100%, with the median diagnosis of CRC being approximately at 35-45 years [3]. Patients with FAP are also at increased risk of other types of cancer, including duodenal/ampullary cancer (1-10%), thyroid cancer (1-12%), gastric cancer (0.5-1.3%), and hepatoblastoma (1-2%) [2].

The management of this disease is based on early screening and timely follow-up, with subsequent planning of risk-reducing or therapeutic surgeries. We present a case of a patient with a strong family history of FAP with a “de novo” diagnosis of CRC. In addition, we conduct a literature discussion of current and future treatment perspectives focusing on colorectal manifestations.

## Case presentation

**Figure 1:**
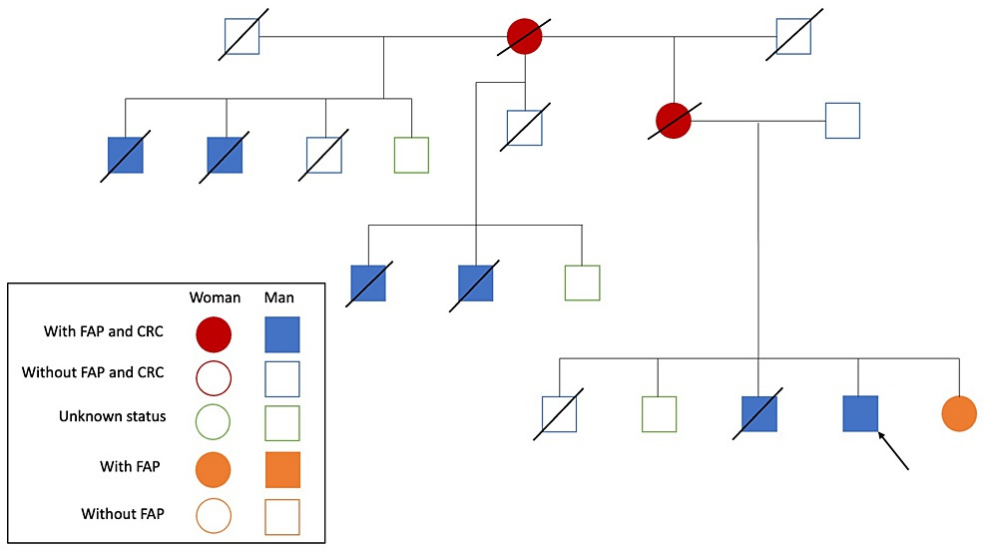
Familial pedigree of multiple cases of familial adenomatous polyposis (FAP) and colorectal carcinoma (CRC)

A 48-year-old man presented to the emergency department for high-intensity abdominal pain in the last two days associated with two episodes of emetic food content and three episodes of greenish diarrhea without blood or mucus. Physical examination revealed generalized pain on deep palpation of the abdomen without the presence of peritoneal signs. He mentions unquantified weight loss in the last six months. Notable in his past medical history is the multiple members of his family with intestinal polyps and colon cancer (Figure 1). Laboratory tests showed hemoglobin levels at 8.5 g/dL (reference range: 12.00-17.00 g/dL) and mean corpuscular volume at 70 fL (reference range: 80.00-100.00 fL), as well as a positive fecal occult blood test. Normal serum values ​​of electrolytes, creatinine, hemoglobin, glucose levels, pH, and lactic acid were found.

**Figure 2:**
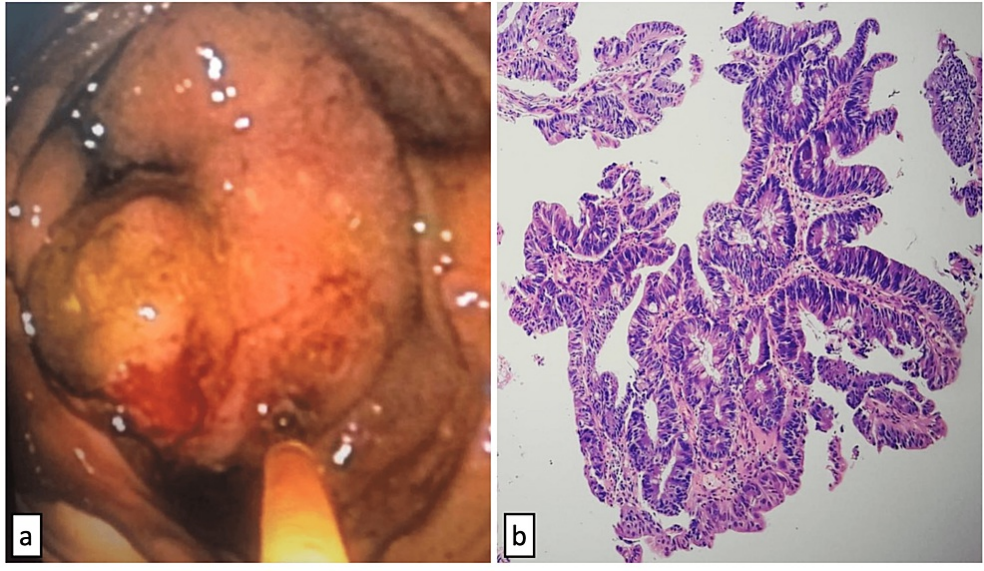
Moderately differentiated usual-type adenocarcinoma of the colon: a) Colonoscopy with a cecum tumor lesion with regular infiltrating edges; b) Histopathologic analysis with colonic epithelium lined by columnar cells with mucin depletion, hyperchromatic nuclei, and frequent mitotic figures

An esophagogastroduodenoscopy (EGD) and colonoscopy were requested, and the EGD was normal. Colonoscopy reported more than 100 polyps in the descending, transverse, and ascending colon, smaller than 5 mm. In addition, a tumor lesion with regular infiltrating edges was demonstrated in the cecum occupying 85-90% of its cross-sectional area. The biopsy of the cecum showed colonic mucosa lined by epithelium with severe dysplasia and desmoplastic reaction, with a final diagnosis of moderately differentiated usual-type adenocarcinoma (Figure 2).

The patient was discharged and followed up by the oncology surgery service for surgery planning. Furthermore, an active search is being carried out for family members for whom CRC screening has not been performed.

## Discussion

The fundamental pillar of FAP management continues to be early screening and timely follow-up. According to international guidelines, patients with "classical FAP" should start a CRC screening program at 10-15 years with follow-up every one-to-two years, with colonoscopy being the procedure of choice [3].

Early screening programs are fruitful if adequate genetic counseling is carried out with the standardization of clinical suspicion criteria. For people with FAP or a family history of it, genetic counseling aims to provide insight into the disease's inheritance pattern to help them make an informed decision about whether or not to accept an offer of genetic testing [1]. Screening recommendations depend on the medical society, where European guidelines suggest that the presence of a personal history of more than 25 colorectal adenomas, a personal history of more than 10 colorectal adenomas before the age of 50, a personal history of three adenomas in those under 30 years of age, a history family of any of the adenomatous polyposis syndromes, or presence of a family history of early-onset CRC are some criteria that suggest the idea of conducting a genetic study (APC and MUTYH gene mutation analysis, being a medium-throughput DNA sequence analysis the gold standard) [4,5]. However, American guidelines recommend performing a genetic evaluation if individuals have a personal history of more than ten cumulative colorectal adenomas, a family history of of the adenomatous polyposis syndromes, or a history of adenomas and FAP-type extracolonic manifestations [5]. 

At the time of FAP diagnosis, surgery is the most offered method to mitigate the risk of CRC. In the majority of cases, a patient is referred for prophylactic surgery if they have an adenoma burden of more than 20 adenomas in the rectum. However, other independent risk factors for a progressive disease include the presence of more than 500 polyps, APC gene mutations between codons 1,250 and 1,450, and age younger than 25 years at the time of surgery [6]. American guidelines provide absolute recommendations to perform immediate colorectal surgery if documented or suspected cancer or significant symptoms and relative indications if there is the presence of multiple adenomas > 6 mm, a significant increase in adenoma number, the presence of an adenoma with high-grade dysplasia, and an inability to adequately survey the colon because of multiple diminutive polyps [5]. Prophylactic surgery options are resection of the colon with an ileorectal anastomosis (IRA) or resection of the colon and rectum with an ileus pouch-anal anastomosis (IPAA) [6]. The risk of developing CRC in patients with previous prophylactic surgery is around 11-24% but this data includes patients before the use of IPAA, in addition to not taking into account the new data from new advanced endoscopic techniques, where especially performing cold loop polypectomy has allowed a comprehensive approach, safely, and with minimal risk of bleeding or perforation [7]. Likewise, the possibility of a surgical procedure with rectal preservation has been studied. Pasquer et al. performed a study with a collection of prospective and retrospective data showing that this approach is possible and safe, even in patients with high-risk characteristics [8].

Another modality that has brought great interest is chemoprevention in patients with FAP, which consists of using drugs to reduce the burden of polyps and delay the time of surgical intervention [9]. An ideal chemopreventive agent would be one that has a mechanism of action related to the pathogenesis of the disease and is safe and easily tolerated over a prolonged treatment period to produce a significant clinical effect [10]. The target studied the most is the inhibition of cyclooxygenase (COX) since prostaglandins play an essential role in the adenoma-carcinoma cascade. The most widely used COX-inhibiting drugs have been sulindac, celecoxib, rofecoxib, fish oil, and aspirin. Multiple studies have shown that these drugs, alone or with a second one, such as difluoromethylornithine (DFMO) or erlotinib, are still ineffective [9-11]. Another signaling pathway of interest has been the mammalian target of rapamycin (mTOR), which is involved in angiogenesis, uncontrolled cellular anabolism, and metastatic transformation [12]. Case series using anti-mTOR drugs activity such as rapamycin, sirolimus, and metformin, also have not given satisfactory results [10,13,14]. Imatinib, a tyrosine kinase inhibitor, has also been used in small surveys showing no clear benefit [15].

## Conclusions

FAP is a genetic disease with an autosomal inheritance that carries a high risk of developing CRC and other types of cancer, including duodenal/ampullary, thyroid, and gastric cancer. The management of this disease is based on early screening and timely follow up, with subsequent planning of risk-reducing or therapeutic surgeries, including less invasive and safe surgical or endoscopic approaches. Chemoprevention in patients with FAP is an alternative to reduce the burden of polyps and delay the time of surgery. However, it is pending the development of studies with solid evidence to use it.
